# Sterile Endophthalmitis after Intravitreal Injections

**DOI:** 10.1155/2012/928123

**Published:** 2012-08-29

**Authors:** Joaquín Marticorena, Vito Romano, Francisco Gómez-Ulla

**Affiliations:** ^1^Retina and Vitreous Unit, Instituto Oftlamológico Gómez-Ulla, 15705 Santiago de Compostela, Spain; ^2^Department of Ophthalmology, Second University of Naples, 81100 Naples, Italy; ^3^Department of Ophthalmology, University Hospital Complex of Santiago de Compostela, 15706 Santiago de Compostela, Spain

## Abstract

Sterile endophthalmitis appears as an infrequent complication of intravitreal injections and seems to develop mainly in the context of the off-label use of drugs that have not been conceived for intravitreous administration. The aetiology of sterile endophthalmitis, independently of the administered drug, remains uncertain and a multifactorial origin cannot be discarded. Sterile inflammation secondary both to intravitreal triamcinolone acetonide and to intravitreal bevacizumab share many characteristics such as the acute and painless vision loss present in the big majority of the cases. Dense vitreous opacity is a common factor, while anterior segment inflammation appears to be mild to moderate. In eyes with sterile endophthalmitis, visual acuity improves progressively as the intraocular inflammation reduces without any specific treatment. If by any chance the ophthalmologist is not convinced by the sterile origin of the inflammation, this complication must be treated as an acute endophthalmitis because of the devastating visual prognosis of this intraocular infection in the absence of therapy.

## 1. Introduction

It was Rycroft in 1945 who first described the intravitreal injection of penicillin for the treatment of endophthalmitis [1]. Intravitreal injections give the opportunity of administering the drug straight where it is necessary. The vitreous cavity offers the great advantage of being a reservoir where high levels of drugs can be maintained for long periods, exceeding by far the concentrations obtained by the administration of drugs through other ways (i.e., topical, intravenous) and minimizing possible systemic side effects due to the small dose given and the little amount of drug that may escape from the eye into the systemic circulation. All these advantages and the presence of novel drugs designed specially for intravitreal use have produced an enormous increase in the number of intravitreal injections administered. The safety profile of intravitreal injections depends not only on the surgical technique, but also on the characteristics of the administered drug. Probably, the most feared and potentially devastating complication of intravitreal injections is endophthalmitis. Once the diagnosis of acute infectious endophthalmitis is suspected, vitreous tap for microbiological study and administration of intravitreal antibiotics must be done, while pars plana vitrectomy will be necessary in a subgroup of patients [2]. Prompt diagnosis and treatment of this entity are crucial for obtaining the best visual prognosis. On the other hand, certain intravitreal-administered therapies can produce an acute and sterile intraocular inflammation that can mimic a true endophthalmitis, but the former is related to good visual prognosis with resolution without the need of intravitreal antibiotics or surgical treatment. For the ophthalmologist it is crucial to know the potential inflammatory reaction that can be associated with the use of certain therapies, as well as to distinguish sterile endophthalmitis from infectious endophthalmitis in order to establish the adequate treatment. The purpose of this paper is to describe the clinical features of sterile endophthalmitis and to discuss the possible mechanisms involved in the development of inflammation after the administration of different drugs by intravitreal injection.

## 2. Definition of Sterile Endophthalmitis

For the purpose of this paper, we have defined sterile endophthalmitis as the acute intraocular inflammation of the vitreous cavity that resolves without the need of intravitreal antibiotics and/or vitreoretinal surgery. Necessarily, if vitreous microbiological study has been done, it needs to be negative culture proven. Patients treated with intravitreal antibiotics or vitrectomy, despite having negative cultures, were excluded from the analysis since an infectious origin of the inflammation cannot be ruled out [2]. The administration of topical antibiotics alone or in combination with intravenous antibiotics was not considered an exclusion criterion for being a sterile endophthalmitis since these treatments would not resolve by themselves a true acute infectious endophthalmitis. A review of the literature published in Pubmed between 1945 and June 2012, searching for keywords endophthalmitis, pseudoendophthalmitis, sterile endophthalmitis, and pseudohypopyon in combination with intravitreal injection, was done. Results were restricted to articles in English and Spanish. The search retrieved 334 articles that were analysed. Other articles referenced in the literature obtained through the initial search were also included.

## 3. Triamcinolone Acetonide

Triamcinolone acetonide is a white-colored, crystalline steroid. Almost insoluble in water, triamcinolone has an anti-inflammatory power 5 times greater than hydrocortisone. Because of the antiangiogenic and antioedematous properties of triamcinolone acetonide, it has been widely used as an off-label treatment for numerous eye diseases that have new vessels or an alteration of the blood-eye barriers. The development of sterile endophthalmitis after intravitreal triamcinolone acetonide (IVTA) has been described by numerous authors [3–12], and it is supposed to occur between 0.20% and 6.73% of the injections [4–7]. However, these numbers need to be interpreted cautiously since most of the reports are based on retrospective studies or small case series; therefore distinguishing sterile endophthalmitis from endophthalmitis can be difficult. Some cases catalogued in the literature as sterile endophthalmitis were treated with intravitreal antibiotics or vitrectomy, making it impossible to discard a true endophthalmitis. Other cases have been catalogued as sterile endophthalmitis just because of negative cultures. In other occasions vitreous haze secondary to dispersion of triamcinolone particles is difficult to differentiate from a real inflammatory process affecting the vitreous [5].

Sterile endophthalmitis secondary to IVTA has been described as a decrease in visual acuity that occurs more frequently within the first 3 days from the injection. Patients usually do not complain of eye pain. Slit-lamp examination may show some signs of mild-to-moderate intraocular inflammation in the anterior chamber such as flare, cells, and keratic precipitates [3, 4, 6]. Usually, hypopyon is not present [3, 4, 10, 11] and fundus examination typically reveals deep vitreous haze obscuring the retina. Nevertheless, it seems necessary to mention that, in the series described by Nelson et al. [5], eye pain was present in 4 cases while 7 cases had a severe inflammatory reaction in the anterior chamber with hypopyon. In the absence of specific treatment, vitreous haze can disappear between 2 weeks to 2 months [3, 4, 6]. Visual prognosis does not seem to be deteriorated and only some few cases have experienced a decrease of visual acuity despite clearing of the media. In these patients, visual decrease was most probably secondary to the underlying pathology than to the temporal inflammatory process.

The aetiology of sterile endophthalmitis is not fully understood. Contamination of triamcinolone vials with endotoxins has been postulated as a possible cause [4]. However, in the context of a cluster of sterile endophthalmitis, no endotoxins were found in the commercial vials of triamcinolone tested [12]. A toxic effect of the triamcinolone itself as well as the preservatives present in the vial (benzyl alcohol, polysorbate 80 and carboxymethylcellulose sodium) has been suggested. Retinal pigment epithelium and glial cells damage [13–15], together with an alteration in the morphology of rabbit photoreceptors, have been observed after the exposure to benzyl alcohol or commercial triamcinolone acetonide given at doses slightly higher than those used in human eyes [16, 17]. On the other hand, other studies in rabbits have not observed signs of cellular toxicity on morphologic or electrophysiologic tests [18–20]. Removal of benzyl alcohol by filtering the commercially available triamcinolone has been proposed as a possible method to reduce the rate of sterile endophthalmitis [9], but a couple of cases have been described even though triamcinolone was filtered and benzyl alcohol almost completely removed before IVTA [11]. An immune response to triamcinolone or any of the preservatives of the commercial vial has been also suggested as a possible cause of sterile endophthalmitis due to the development of intraocular inflammation after a second intravitreal injection [4]. Allergic reactions to triamcinolone have been described, but most possibly these cases corresponded to a reaction to any of the preservatives [21–23]. We observed a repeated episode of sterile endophthalmitis in a patient treated in 2 consecutive occasions with combined photodynamic therapy with verteporfin and IVTA [10]. In that patient, systemic and cutaneous allergic tests were negative; therefore, hypersensitivity reaction type 1 and type 4 were ruled out. However, non-IgE-mediated reactions have been observed with polysorbate 80. Considering that there are no systemic allergic reactions that would be necessary to prevent in patients with sterile endophthalmitis secondary to IVTA, the performance of allergy tests is of doubtful utility. Furthermore, negative allergy tests do not discard a future episode of inflammation.

## 4. Pseudoendophthalmitis and Pseudohypopyon after Triamcinolone Acetonide

Pseudoendophthalmitis is an infrequent complication of IVTA and occurs in about 0.74–0.8% of the injections [6, 24]. The term pseudoendophthalmitis has been used previously as synonymous of sterile endophthalmitis, but most of the authors use it to the describe the dispersion of triamcinolone crystals and their passage from the vitreous cavity to the anterior chamber [25], more frequently in eyes with posterior capsule impairment or suspected zonular defect after being vitrectomized [8, 24, 26–29]. The settling of the crystals in the inferior angle of the anterior chamber produces the appearance of a “pseudohypopyon.” This has been observed to happen immediately after the intravitreal injection, but usually occurs within the first 3 days. Patients typically do not present eye pain, conjunctival hyperemia, or any sign of intraocular inflammation [6, 24, 26, 29–34]. Pseudohypopyon usually can be differentiated from true inflammatory hypopyon on the slit lamp. Chen et al. [26] recommend to distinguish pseudohypopyon from true inflammatory hypopyon by tilting the patient's head and observing the shifting of the crystals upon the new position. Despite the amount of triamcinolone occupying the angle, no changes in the intraocular pressure have been associated with pseudohypopyon. Washout of the anterior chamber has been described in two cases of high-dose IVTA injections [29, 33], while all other cases resolved spontaneously between 4 days and 2 months [6, 24, 27, 30–32, 34]. No alterations of the anterior segment structures have been described once the triamcinolone reabsorbed.

## 5. Antivascular Endothelial Growth Factor**** Drugs

Bevacizumab (Avastin, Genentech, Inc., San Francisco, California, USA) is a full-length humanized monoclonal nonselective antibody against vascular endothelial growth factor approved by the Food and Drug Administration for the treatment of glioblastoma and of metastatic colorectal cancer, advanced nonsquamous non-small-cell lung cancer and metastatic kidney cancer in combination with chemotherapy. Rosenfeld et al. described for the first time the use of intravitreal bevacizumab (IVB) for the treatment of macular oedema secondary to retinal vein occlusion and exudative age-related macular degeneration [35, 36]. Since then, several studies have described the off-label use of IVB for the treatment of numerous vascular and oedematous eye diseases. The incidence of acute cultured proven endophthalmitis appears to be very low, ranging from 0.02% to 0.16% [37–39], while the incidence of sterile endophthalmitis has been described between 0.09% and 1.1% of IVB injections [37, 40–43].

An early and acute decrease in visual acuity appears as the most common symptom in patients with sterile endophthalmitis secondary to IVB. This can occur during the first 48 hours after the intravitreal injection and in all cases seems to be within the first week [40–42, 44]. Despite the intraocular inflammation, ocular pain seems to be infrequent [42]. Of the 44 cases observed by Chong et al. [40] blurred vision was present in 73% of the patients, floaters in 43%, and pain in 34%. Most of the patients had signs of inflammation in vitreous cavity (80%) as well as in the anterior chamber (77%). Considering just those eyes of this series that presented signs of inflammation that did not receive intravitreal antibiotics/vitrectomy, inflammation was mild to moderate in the anterior chamber in 7 out of 9 cases and mild to moderate in the vitreous cavity in 8 out of 9 cases. Interestingly, Georgopoulos et al. [42] observed a “pseudogranulomatous” inflammation of the vitreous because of the presence of large cellular aggregates. None of the reported cases with sterile inflammation presented fibrin or hypopyon [40–42, 44]. In the internet-based survey done by Fung et al. [41] all 10 cases of inflammation were catalogued as mild or moderate and lasted no longer than a week while sterile endophthalmitis cases reported by Chong et al. [40] resolved after 37 ± 5 days. These authors observed that mean time for visual acuity recovery was 53 ± 18 days and there was no difference between visual acuity observed at the end of the inflammatory process compared with pretreatment visual acuity [40]. A similar situation was observed in the 8 cases described by Georgopoulus et al. [42] where all patients but one recovered initial visual acuity.

It is necessary to mention that different degrees of acute anterior segment inflammation have been described after 0.25% of IVB injections [45]. Sterile intraocular inflammation has been described in patients with a severe inflammatory reaction in the anterior segment of the eye. Ocular pain and hypopyon were present in some of these patients, whereas vitreous inflammation was mild to moderate [46–49].

Diverse hypotheses have been proposed to explain the inflammatory response secondary to IVB. The solution of bevacizumab for intravenous administration comes in vials of 100 mg/4 mL or 400 mg/16 mL; therefore, obtaining different 0.1 mL or 0.05 mL doses for intravitreal use implies the manipulation and possible risk of contamination of the solution. As Wickremasinghe et al. mentioned in their report [43], although contamination of individual aliquots of bevacizumab with bacterial endotoxins during preparation may occur, this theoretical situation could explain clusters of sterile endophthalmitis in patients treated with injections coming from the same batch [44], but seems unlikely to be the cause of sporadic cases. Bacterial endotoxins are frequent and recalcitrant contaminants of antibody preparations during the production phase of the drug [50]. Preparations of bevacizumab that are originally designed for intravenous use may contain traces of endotoxin at levels that incite intravitreous inflammation, even though they are of no significance when the drug is administered systemically [43]. A specific immune reaction to the anti-VEGF antibody could also explain the development of sterile inflammation. Different authors have highlighted the presence of sterile endophthalmitis after repeated intravitreal bevacizumab injections [40, 43]. However, sterile endophthalmitis can develop after the first IVB. Another important fact is that the manufacture of bevacizumab recommends to keep it refrigerated between 2 and 8°C and protected from light [51]. Fluctuation of the temperature has been proposed as a factor that may increase immunogenic properties of bevacizumab [43]. Temperature fluctuation has been demonstrated to increase the immunogenicity of therapeutic proteins [52]. This may be due to protein degradation creating novel antigenic epitopes not found in the parent molecule [53].

Ranibizumab (Lucentis, Novartis Pharma AG; Genentech USA Inc.) is a recombinant, humanized monoclonal antibody Fab that neutralises all active forms of VEGF-A. Ranibizumab is approved for the treatment of exudative age-related macular degeneration, diabetic macular oedema, and macular oedema secondary to retinal vein occlusion. Pseudoendophthalmitis was reported to occur in 1 out of 599 patients (0.16%) treated with ranibizumab in the CATT study [54]. Unfortunately, there is no detailed information regarding the characteristics of this episode. Fauser et al. described 2 consecutive episodes of intraocular inflammation in the same patient. It occurred 24–48 hrs after the injection and visual acuity decrease, eye pain, hypopyon, and moderate vitreous cells were present [55]. The first episode was treated with intravitreal antibiotics, but no specific treatment was given for the second episode. Interestingly, there was no recurrence of the inflammation after a subsequent injection of ranibizumab. Sharma et al. described 1 patient (1/891 injections, 0.11%) with mild anterior chamber inflammation together with mild vitritis 3 days after ranibizumab injection [56]. There was spontaneous resolution of the inflammation and improvement of visual acuity. As far as the authors are aware, there are no other cases in the literature describing the development of sterile endophthalmitis secondary to intravitreal ranibizumab. The very low frequency of this adverse event may be related to the characteristics of the molecule, but the ultimate cause remains to be elucidated.

## 6. Methotrexate

Sterile endophthalmitis has been also described after the intravitreal injection of methotrexate in patients with primary central nervous system lymphoma involving the eye. Usually, multiple intravitreal injections of 200–400 *μ*g/0.1 mL of methotrexate are required to observe the remission of the disease. In the literature there are some few cases of acute intraocular inflammation that developed after intravitreal methotrexate, but the majority of these cases lack detailed description of the ocular signs and evolution. In a series of 16 patients treated with intravitreal methotrexate, 1 patient developed intraocular inflammation that was catalogued as sterile endophthalmitis [57]. Microbiologic cultures were negative and the inflammation remitted rapidly after the administration of intravitreal antibiotics in combination with topical and systemic corticosteroids. In another series of 44 eyes from 26 patients, 2 patients developed severe intraocular inflammation that responded to topical steroids; the first one was catalogued as sterile endophthalmitis while the second was assumed as a toxic anterior segment syndrome [58]. However, it is important to mention that this entity is characterized by an early and intense postoperative inflammation after anterior segment surgery accompanied by minimal or no pain, fibrin formation, corneal edema, and the absence of vitreous involvement [59]. The mechanism of inflammation after intravitreal methotrexate remains uncertain.

## 7. Conclusions

Sterile endophthalmitis appears as an infrequent complication of intravitreal injections and seems to develop mainly in the context of the off-label use of drugs that have not been conceived for intravitreous administration. Sterile inflammations secondary to IVTA and to IVB share many characteristics such as the acute and painless vision loss present in the big majority of the cases. Dense vitreous opacity is a common factor, while anterior segment inflammation appears to be mild to moderate. Hypopyon is a very infrequent sign in the context of sterile inflammation after intravitreal injections. In eyes with sterile endophthalmitis, visual acuity improves progressively as the intraocular inflammation reduces without any specific treatment. In this study eyes treated with intravitreal antibiotics or vitrectomy were not included. This may constitute a bias by excluding severe cases that presented signs such as ocular pain or hypopyon. If by any chance the ophthalmologist is not convinced by the sterile origin of the inflammation, this complication must be treated as an acute endophthalmitis because of the devastating visual prognosis of this intraocular infection in the absence of therapy. The aetiology of sterile endophthalmitis, independently of the administered drug, remains uncertain and a multifactorial origin cannot be discarded.
